# Treatment outcomes among children admitted stabilization centers in Eastern Ethiopia: retrospective study

**DOI:** 10.3389/fpubh.2023.1165858

**Published:** 2023-07-18

**Authors:** Jemal Abrahim Ahmed, Newas Yusuf, Tara Wilfong, Kedir Negesso Tukeni, Hiwot Berhanu, Kedir Teji Roba

**Affiliations:** ^1^School of Public Health, Haramaya University, Harar, Ethiopia; ^2^College of Health Sciences, School of Public Health, Haramaya University, Harar, Ethiopia; ^3^Department of Internal Medicine, Jimma University, Jimma, Ethiopia

**Keywords:** treatment outcome, SAM with complication, east Hararghe, Ethiopia, retrospective study

## Abstract

**Background:**

There is improved access to Sever Acute Malnutrition management in Ethiopia; however, studies have revealed an alarming rate of defaulters’ poor recovery and deaths, emphasizing the importance of researching to identify major causes. As a result, the goal of this research is to identify treatment outcome determinants and associated factors in severely malnourished children aged 6–59 months admitted to public hospitals in Eastern Ethiopia’s stabilization centers.

**Methods:**

This study used an institutional-based retrospective cohort study design with 712 children aged 6 to 59 months. Data was gathered using a Sever Acute Malnutrition registration logbook and patient charts. Participants were chosen at random from their respective healthcare facilities based on population proportion. Epi-data was entered and analyzed using STATA version 14. To identify associated factors, the Cox proportional hazard Ratio was calculated, and a *p*-value of 0.05 at the 95% confidence interval was considered statistically significant.

**Results:**

This study revealed that only 70.65% (95% CI = 67.19, 73.88) of the children were cured while 17.84% defaulted from the management and 5.90% died. Children who did not have tuberculosis (AHR = 1.58, 95%CI:1.04, 2.40), anemia (AHR = 1.31, 95% CI:1.03, 1.68), Kwash dermatosis (AHR = 1.41, 95%CI:1.04, 1.91), or on NG-tube (AHR = 1.71, 95%CI:1.41, 2.08) were more likely to be cured from SAM.

**Conclusion:**

This study discovered that the cure rate is extremely low and the defaulter rate is extremely high. As a result, intervention modalities that address the identified factor are strongly recommended to accelerate the rate of recovery in Eastern Ethiopia.

## Introduction

Severe acute malnutrition (SAM) is a nutritional deterioration identified by anthropometric indicators such as a WH/L -3Z score or MUAC 11.5 cm and/or the presence of nutritional edema (1, 2). Malnutrition remains a major public health issue worldwide, with 149.2(22%) million under-five children stunted, 45.4(6.7%) million under-five children wasted, and 38.9(5.7%) million children undernutrition. According to studies, more than half of all wasted children live in Southern Asia (SA) and Sub-Saharan Africa (SSA). In Africa, more than 12 million under-five children were wasted, of which 3 million were SAM (3). In addition, according to Ethiopian Mini-Demographic and Health Survey 2019 (Mini-EDHS 2019) reported that 7% of under-five children are wasted (4).

Even though SAM is a preventable cause of under-five children mortality and morbidity, children with SAM were nine times more likely to die compared to their counterparts (5). Thus, SAM is responsible for the deaths of 3.6 million children under the age of five worldwide, with 45–60% of these deaths occurring in low- and middle-income countries (LIMC) (6). Despite advances in child health and nutritional interventions (7), undernutrition was responsible for more than 28% of child deaths in Ethiopia (8).

Children with SAM and medical complications such as clinical signs of infection, severe edema and or anemia, metabolic disturbance, vomiting, hypothermia, dehydration, and poor appetite must be admitted to a stabilization center, according to the WHO protocol. According to the guidelines, they are primarily treated with F75 during stabilization and F100 or ready-to-use therapeutic foods (RUTF) during the transition, as well as other standard medications such as antibiotics, deworming, folic acid, and vitamin A.

A recent systematic review conducted in SSA and Ethiopia found that the recovery rate from SAM remains as low as 71.2 and 72.02%, respectively (9, 10). Other pocket studies in Ethiopia reported that the recovery rates ranged from 58.4% in the Bahir Dare referral Hospital to 70.4% in Yekatit 12 Ethiopian Hospitals (6, 11–15). Similarly, previous studies in Ethiopia also discovered that children’s socio-demographic and economic factors and baseline anthropometric characteristics, medical-related factors, adherence to the treatment protocol, duration of follow-up, and types of comorbidity on admission were the underlying determinants of treatment recovery (6, 11–15). However, to the knowledge of these researchers, there are no published studies that assessed the recovery rates from two regions of eastern Ethiopia among children admitted to stabilization centers. As a result, the main objective of this study was to identify the recovery rate and associated factors among under-five children with SAM admitted to public hospitals in Eastern Ethiopia’s stabilization centers.

## Materials and methods

### Study settings and period

This study was carried out at two public hospitals in East Hararghe Zone, Haramaya and Chelenko hospitals, as well as Hiwot-Fana specialized hospital in Harari Regional state, from December 10 to 30, 2021. Harar, located 525 kilometers east of Addis Abeba, served as the regional capital city of Harari as well as the East Hararghe Zone’s Zonal Administrative Center. Eastern Hararge zone is the most populous of the Oromia regional state’s 24 zones, with a total population of 3.7 million (16). There are six public hospitals, 121 health centers, and 548 health posts in this zone. The Harari Region of Ethiopia is one of nine regional states. There are a total of 232,000 people, with 116,928 men and 115,072 women. One military hospital, two public hospitals, one private hospital, and eight health centers are available.

### Study design and population

An institutional retrospective cohort study was conducted among children aged 6 to 59 months who were admitted to randomly selected hospitals. The study population consisted of records of children aged 6–59 months admitted to stabilization centers in public hospitals in East Hararghe and Harari Region with the diagnosis of SAM. The study excluded records with missing or incorrect information.

### Sample size and sampling procedure

The sample size was determined using Epi-info, Version 7.2, under the following assumptions: This study used anemia on admission as an exposure variable, with a 95% CI and a power of 80%. The percentages of recovery among the unexposed-to-anemia and exposed-to-anemia groups were 75.42 and 63%, respectively. Then a one-to-one ratio of unexposed to exposed, a 1.5 design effect, and 15% accounting for missing or incomplete data (11). As a result, the final sample size was 744.

In this study, a two-stage cluster sampling technique was used. All public hospitals in East Hararge Zone and Harari Regional State provide SAM inpatient care. Haramaya and Chelenko were chosen from the six East Hararge Zones of Oromia Regional State, and Hiwot-Fana specialized university hospital was chosen from two public hospitals in Harari Regional State. Each hospital had its sampling frame, and the registration log book was used to identify eligible SAM patients admitted to the chosen hospitals. Based on the number of patients seen in the previous 6 months, each hospital received a sample. Finally, eligible study subjects were chosen from each hospital using a systematic random sampling technique.

### Data collection tools and methods

The checklist was created by combining the standard SAM management treatment protocol, the registration log book, and the SAM monitoring multi-chart. The checklist includes socio-demographics anthropometry (MUAC, weight, and height/length), presences or absences of edema, immunization status, medical diagnosis at admission, and treatment outcome of severe acute malnutrition were collected among the others (2) (Attached as supplement at the end of this manuscript).

Two supervisors and five data collectors (with BSc clinical Nurse practitioners) with SAM management experience were hired and given 2 days of training on data collection tools. The tool was tested on 5% of the sample size at a nearby public hospital with a comparable population before being included in the study. The pretest findings were discussed among supervisors, data collectors, and the principal investigator to ensure a better understanding of tools and procedures, and the final version was modified as a result. During data collection, the SAM registration log book, the children’s cards or folder, and the SAM monitoring multi-chart were all reviewed. Until the final data collection day, consistency and completeness were checked daily.

### Data processing and analysis

The data were double entered EPI Data after being cleaned, coded, and loaded into the Epi Data version 3.1 software and were exported and analyzed using STATA version 14.2 software. The study’s independent variables were described using descriptive statistics such as frequency distribution and percentage. To determine the relationship between the recovery rate and each independent variable, bivariable and multivariable Cox regression analyses were performed. Before running the Cox-proportional hazard, assumption tests were conducted for each variable using a log–log plot and the Schoenfeld residuals test (global test).To account for potential confounders, all variables with *p*-values less than 0.25 in the bivariable Cox regression analysis were identified and included in the multivariable model. The Cox-Snell plot was used to evaluate the overall fitness of the model. Finally, the AHR 95% confidence interval estimation was used to identify factors associated with recovery from severe acute malnutrition. The variable that shows statistical significance (*p*-vales < 0.05) in the multivariable analysis was considered statistically significant.

## Results

### Socio-demographic and character of the children at admission

In this study, 712 children’s records were included in the analysis from a total sample size of 744, with a completeness rate of 96% and 32 records being incomplete and excluded. The majority of the children (55.34%) were males between the ages of 12 and 23 months (median age = 18 months), with 353 (49.58%) breastfeeding at the time of admission. 625 (87.78%) of the total study participants were admitted as new cases of SAM children, with 513 (72.05%) referred from a health facility. Malnutrition was classified as marasmus, Marasmic-Kwashiorkor, or kwashiorkor in 420 (58.99%), 163 (22.89%), and 129 (18.12%) cases, respectively. The mean weight and MUAC of children during admission were 7.12 kg (SD 2.25) and 11.25 kg (SD 1.31), respectively (Table 1).

**Table 1:** Socio-demographic and characters of the children aged 6–59 months admitted to SC of selected public hospitals in East Hararghe Zone and Harari Region, Ethiopia.

Characteristics	Frequency (*n* = 712)	Percentage (%)
Age of child in months		
6–11	168	23.60
12–23	214	30.06
24–35	170	23.88
36–59	160	22.47
Sex		
Male	394	55.34
Female	318	44.66
Place of residence		
Urban	168	23.60
Rural	544	76.40
Vaccination status		
Fully Vaccinated	266	37.36
Partially Vaccinated	296	41.57
Not Vaccinated	150	21.07
Admission status		
New	625	87.78
Re-admission	87	12.22
Referral Source		
Health facility	513	72.05
Self-Referred	199	27.95
Breast Feeding on Admission		
Yes	353	49.58
No	359	50.42
Appetite test at admission		
Fail	634	89.04
Pass	78	10.96
Admission criteria		
Only wasting (Marasmic)	420	58.99
Only edema (Kwashiorkor)	129	18.12
Marasmic-kwashiorkor	163	22.89
WFH/L		
< −3Z-score	469	65.87
≥ −3Z-score	243	34.13
MUAC		
<11.5 cm	440	61.8
≥ 11.5 cm	272	38.2
Type of malnutrition		
Edematous	292	41.01
Non-edematous	420	58.99

### Routine medication and treatment

Ampicillin, gentamycin, and ceftriaxone were administered intravenously to nearly all of the admitted children (97.05%). Furthermore, F-75 therapeutic milk was given to 89.33% of malnourished children, 74.02% were given F-100, and 208 (29.21%) were given plumpy nuts. Following admission, 37.64% of all admitted children were given Vitamin A supplements, and 40.45% were given Folic acid supplements. In comparison, only 5.34% of all children received blood during inpatient treatment, and 3.65% were resuscitated with IV fluid during inpatient waiting time. Almost half of SAM children (43.26%) have an NG tube during treatment. While 30.20% of the children were dewormed following their admission (Table 2).

**Table 2:** Medication provision and mineral supplementation of SAM children aged 6–59 months admitted to SC of selected public hospitals in East Hararghe Zone and Harari Region, Ethiopia.

Variables	Frequency (*n* = 712)	Percent (%)
Vitamin A		
Yes	268	37.64
No	444	62.36
Folic acid		
Yes	288	40.45
No	424	59.55
Deworming		
Yes	215	30.20
No	497	69.80
Oral-Antibiotics		
Yes	207	29.07
No	505	70.93
ReSomal		
Yes	419	58.85
No	293	41.15
IV-fluid		
Yes	26	3.65
No	686	96.35
IV-antibiotics		
Yes	691	97.05
No	21	2.95
Blood transfusion		
Yes	38	5.34
No	674	94.66
Intake of F75		
Yes	636	89.33
No	76	10.67
Intake of F100		
Yes	527	74.02
No	185	25.98
Plumpy’nut		
Yes	208	29.21
No	504	70.79
Feed by		
Orally	404	56.74
NG-tube	308	43.26

### Medical comorbidity

The majority (95.79%) of children admitted to the stabilization center had at least one type of co-morbidity while 44.38% of children were admitted with three or more. Among those with diagnosed comorbidity, 96.93% had marasmus-kwashiorkor, 97.38% had marasmus, and 89.15% had kwashiorkor at the time of admission. The most common co-morbidities were pneumonia (44.10%), diarrhea (62.64%), and anemia (18.68%; Table 3).

**Table 3:** Distribution of medical co-morbidity information on treatment outcomes of 6 to 59 months children with SAM admitted to SC of selected public hospitals in East Hararghe Zone and Harari Region, Ethiopia.

Variables	Frequency (*n* = 712)	Percent (%)
Comorbidity		
Yes	682	95.79
No	30	4.21
Diarrhea		
Yes	446	62.64
No	266	37.36
Vomiting		
Yes	112	15.73
No	600	84.27
Fever (body temp ≥37.5°C)		
Yes	93	13.06
No	619	86.94
Pneumonia		
Yes	314	44.10
No	398	55.90
Hypoglycemia		
Yes	20	2.81
No	692	97.19
Anemia		
Yes	133	18.68
No	579	81.32
Congestive Heart Failure		
Yes	23	3.23
No	689	96.77
Dehydration		
Yes	204	28.65
No	508	71.35
Shock		
Yes	25	3.51
No	687	96.49
Tuberculosis		
Yes	43	6.04
No	669	93.96
HIV/AIDS		
Yes	3	0.42
No	709	99.58
Kwash dermatosis		
Yes	74	10.39
No	638	89.61
Number of Co-morbidities at a time		
No	30	4.21
One	158	22.19
Two	208	29.21
≥three	316	44.38

### Treatment outcomes

In this study, the recovery rate was 70.65% (95% CI: 67.19, 73.88), the death rate was 5.90% (95% CI: 4.39, 7.89), and the default rate was 17.84% (95% CI: 15.19, 20.83). Among those who recovered within 41 days, the median time to recovery was 11 days (95% CI:10, 12), and the incidence of recovery was 8.4 (95% CI: 7.6, 9.1) per 100 person-day observations. The average (SD) weight gain of a severely malnourished child was 13.65(9.2) g/kg/day. The mean (standard deviation) length of stay for recovered edematous and non-edematous children was 12.3(5.98) days and 11.6(5.47) days, with no statistically significant difference [*p* = 0.5626; 220 (75.34%)]. The overall performance of the hospitals, as well as their SPHERE indicators, are shown below (Table 4).

**Table 4:** Performance indicator of stabilization center of selected public hospitals in East Hararghe Zone and Harari Region, as compared to sphere project reference values/international standard.

Indicators	Hiwot-Fana Referral hospital	Haramaya Hospital	Chelenko Hospital	Overall	^a^SPHERE standards	
					Acceptable	Alarming
Recovered (%)	68.03	75.23	69.59	70.65%	>75%	<50%
Defaulted (%)	22.88	9.91	18.71	17.84%	<15%	>25%
Died (%)	7.21	4.95	4.68	5.90%	<10%	>15%
Length of stay				11 days	<28 days	>42 days

### Factors associated with treatment outcomes of severe acute malnutrition

In this analysis, 13 variables with *p*-values less than 0.25 were chosen for multivariable Cox regression. Referral source, vaccination status, admission status, vitamin A administration, fever, pneumonia, anemia, folic acid administration, tuberculosis, having an NG-tube during admission, dehydration during admission, presence with shock, and having Kwash dermatosis on admission are all included. After controlling for potential confounding effects on the outcome variable, factors such as the presence of anemia and tuberculosis during admission, Kwash dermatosis, and NG-tube placement during admission remained significantly associated with treatment recovery. As a result, children who did not have TB infection were 1.58 (AHR = 1.58, 95%CI: 1.04, 2.40) times more likely than those who did recover from SAM. Children aged 6–59 months who did not have an NG-tube during their admission had a 1.71 times higher chance of completing treatment than children who did (AHR = 1.71, 95%CI:1.41, 2.08). Furthermore, children who did not have anemia had a 1.31 (AHR = 1.31, 95% CI:1.03, 1.68) higher chance of recovering from SAM than those who did. Similarly, children who did not have Kwash dermatosis at the time of admission were 1.41 (AHR = 1.41, 95%CI:1.04, 1.91) times more likely than children who did recover from SAM (Table 5).

**Table 5:** Bivariable and multivariable Cox-regression analysis of factors associated with recovery among children admitted with SAM to SC of selected public hospitals in East Hararghe Zone and Harari Region, Ethiopia.

Variables	Recovered (*n* = 503)	Censored (died, defaulter, and transfer out) (*n* = 209)	CHR (95%CI)	AHR (95%CI)
Referral Source				
Health facility	366(72.76)	147(70.33)	0.84(0.69,1.02)	0.87(0.72,1.07)
Self-referral	137 (27.24)	62 (29.67)	1	1
Admission status				
New	447(88.87)	178(85.17)	1.33(1.01,1.75)	1.19(0.89,1.59)
Re-admission	56(11.13)	31(14.83)	1	1
Vaccination status				
Fully vaccinated	212(42.15)	54(25.84)	1.36(1.04,1.77)	1.26(0.96,1.65)
Partially vaccinated	216(42.94)	80(38.28)	1.20(0.92,1.56)	1.11(0.85,1.46)
Not vaccinated	75(14.91)	75(38.28)	1	1
Fever (body temp ≥ 37.5°C)				
Yes	57(11.33)	36(17.22)	0.83(0.63,1.09)	0.94(0.71,1.25)
No	446(88.67)	173(82.78)	1	1
Pneumonia				
No	287(57.06)	111(53.11)	1	1
Yes	216(42.94)	98(46.89)	0.82(0.69,0.98)	0.87(0.73,1.05)
Anemia				
No	423(84.10)	156(74.64)	1.52(1.19,1.93)	1.31(1.03,1.68)
Yes	80(15.90)	53(25.36)	1	1
Tuberculosis				
No	477(94.83)	192(91.87)	1.94(1.30,2.88)	1.57(1.04,2.40)
Yes	26(5.17)	17(8.13)	1	1
Dehydration				
No	373(74.16)	135 (64.59)	1	1
Yes	130(25.84)	74 (35.41)	0.86(0.70,1.05)	0.93(0.75,1.16)
Shock				
No	489(97.22)	198(94.74)	1	1
Yes	14(2.78)	11(5.26)	0.55(0.32,0.94)	0.73(0.41,1.30)
Kwash dermatosis				
No	447(88.87)	191(91.39)	1.32(0.99,1.74)	1.41(1.04,1.91)
Yes	56(11.13)	18(8.61)	1	1
Vitamin A				
No	262(52.09)	182(87.08)	1	1
Yes	241(47.91)	27(12.92)	1.21(1.01,1.44)	0.97(0.62,1.51)
Folic acid				
No	248(49.30)	176(84.21)	1	1
Yes	255(50.70)	33(15.79)	1.19(1.01,1.42)	1.19(0.78,1.85)
NG-tube				
No	319(63.42)	85(40.67)	1.90(1.58,2.29)	1.72(1.41,2.08)
Yes	184(36.58)	124(59.33)	1	1

HIV = human immune-deficiency virus, TB = tuberculosis, NG-tube = naso-gastric tube.

## Discussion

According to this study, 70.65% of children recovered, which falls short of the minimum acceptable international SPHERE standards (>75%) (1). However, the recovery rate in this study was similar to that of Yekatit 12 Hospital Medical College (70.4%) (15) and Lacor Hospital of Uganda (70.6%) (17), while higher than that of Bangladesh (55.7%) (18), Sudan (57.4%) (19), Nigeria (48.5%) (20). In contrast, the recovery rate of this study was lower than that of India (81%) (21), Wag Himra Zone, Northeast Ethiopia (80.4%) (22), Yirgalem Hospital (78%) (23), and Yekatit 12 Hospital (81.3%) (24). The reasons for the low recovery rate in this study compared to other studies could be attributed to an unacceptable higher defaulter rate, differences in socioeconomic status, quality of care provided for children, health-seeking behavior, and the availability and accessibility of therapeutic foods and medications.

Similarly, the default rate of this study was 17.84%, which was higher than the minimum acceptable international SPHERE standards (15%) (1). However, the default rate in this study was in line with Gondar University Comprehensive Specialized Hospital’s 17.79% (11), while higher than Zambia’s 17.2% (17), Pawe general hospital’s 16.52% (6), and Wag Himra Zone, Amhara National Regional State’s 8.2% (22). In contrast, the default rate of this study was lower than that of Bangladesh, which was 29.9% (18), Bahir Dar Felege Hiwot Referral Hospital, which was 21.7% (13), and Ayder referral hospital, which was 43.6% (25). Differences in study sitting, socioeconomic status, quality of care provided for children, health-seeking behavior, and availability and accessibility of therapeutic foods and medications are all possible reasons for the higher default rate in this study.

This study’s participants’ overall median nutritional recovery time was 11 days (IQR: 9–13). The findings are consistent with those of studies conducted in Pawe General Hospital, North West Ethiopia (6), Gonder referral hospitals (11), and Wag Himra Zone, Northeast Ethiopia (22), but they are lower than those of Pawe General Hospital (26), Hawassa Specialized (12). The disparity could be explained by differences in study settings, as some studies were conducted in referral and specialized hospitals where children with the most severe SAM cases are referred.

The presence of other comorbidities at admission greatly influences the prognosis of SAM. The humeral and cell-mediated immunity of children affected by SAM may be depressed and unable to protect children from infection, which may be attributable to the high prevalence of infection. In this study, for example, children who did not have tuberculosis were 1.58 times more likely to recover from SAM than those who did. This study was consistent with research done at Gondar Comprehensive Specialized Hospital (11), Benishangul-Gumuz, Pawi General Hospital (26), Jimma University (27), and Southern Ethiopia (12). Weight loss decreased appetite, and nutrient absorption may occur as a result of tuberculosis infection. This suggests that children with tuberculosis require a longer hospital stay because they have a greater nutritional crisis and require more nutrients than their peers (28).

In this study, we discovered that SAM children who did not have anemia had 1.31 times the chance of recovery as those who did. This finding was consistent with those from the WagHimra Zone in northeast Ethiopia (22), Jimma University Medical Center (27), Gondar Comprehensive Specialized Hospital (11), Nekemte Referral Hospital (14), and Pawi General Hospital (26). This is due to an increase in the prevalence of infection and an increased risk of heart failure in anemic children, resulting in a long time to cure (2).

According to a study conducted at Gondar, children without kwash-dermatosis had a higher probability of recovery (11). This is consistent with our study findings. The possibility is that children with kwash-dermatitis were more prone to infection and metabolic complications, as well as being edematous with more skin lesions, which led to more complications and a longer time to cure and poor appetites.

According to this study children without an NG tube had more likely to recover. A previous study also indicated that children admitted with complicated SAM and who were unable to feed orally were fed via NG tube were more at risk of poor treatment outcomes (29). This result was comparable to the findings of a study conducted in North Wet Ethiopia (6). The presence of an NG tube during admission may result in complications such as diarrhea, vomiting, lung aspiration, and electrolytic alterations, which will reduce the treatment cure rate (30).

The study’s limitations included the difficulty of determining the reliability of recorded data due to the nature of secondary data, as well as failure to address variables such as educational status, household wealth index, socioeconomic status, household family size, and child’s feeding practice that may affect treatment outcome.

### Conclusion

This study found that the recovery rate was low with higher defaulter rate compared to the sphere standard guideline. Thus, prompt and timely management of tuberculosis, anemia, and kwash-dermatosis should be prioritized, as should reducing the use of NG tubes for feeding and medications after admission. Finally, we recommend additional research to determine the cause of this study’s low cure rate and high defaulter rate.

## Data availability statement

The original contributions presented in the study are included in the article/Supplementary material, further inquiries can be directed to the corresponding author.

## Ethics statement

This study’s methods were all carried out following the Helsinki Declaration-Ethical principle for medical research involving human subjects. Haramaya University’s Institutional Research Ethics and Review Committee (IRERC) provided an ethical approval letter with the reference number IHRERC/102/2021. Heads of hospitals provided informed written voluntary signed consent. Because the study was conducted through a review of records, no consent from study subjects is required. All personal identifiers were removed, and data was kept private and only used for the proposed study.

## Author contributions

All authors made significant intellectual contributions to the study’s conception and design, as well as data acquisition, analysis, and interpretation. JA wrote the manuscript, which KR and TW reviewed for intellectual content. All authors contributed to the article and approved the submitted version.

## Conflict of interest

The authors declare that the research was conducted in the absence of any commercial or financial relationships that could be construed as a potential conflict of interest.

## Publisher’s note

All claims expressed in this article are solely those of the authors and do not necessarily represent those of their affiliated organizations, or those of the publisher, the editors and the reviewers. Any product that may be evaluated in this article, or claim that may be made by its manufacturer, is not guaranteed or endorsed by the publisher.

## Abbreviations

AHR: Adjusted hazard ratio
CHR: Crude hazard ratio
CI: Confidence interval
EDHS: Demographic and Health Survey
FMOH: Federal ministry of health
HIV/AIDS: IV, intravenous
MUAC: mid-upper arm circumference
RUTF: Ready-to-use therapeutic foods
SAA: Sub-Saharan Africa
SA: Southern Asia
SAM: Severe acute malnutrition
SC: Stabilization center
SD: Standard deviation
NG: Nasogastric
WFH/L: Weight for height/length
WHO: World Health Organization
