# Supplementary material for: Treatment outcomes among children admitted stabilization centers in Eastern Ethiopia: retrospective study
Source: Front Public Health. 2023 Jul 18;11:1165858. doi: 10.3389/fpubh.2023.1165858 (PMC10392950; doi:10.3389/fpubh.2023.1165858)
Supplement: Supplementary file 1 [file Table_1.docx]

**Supplement I**

**DATA EXTRACTION FORMAT**

Date of review __/___/_____Hospital Name____________________

Available Data: I. Complete____ II. Incomplete_____ III. Excluded____

**Part I. patient baseline Information (filled from therapeutic registration book)**

| **SECTION 1. DEMOGRAPHIC CHARACTERISTICS** | | | | | | |
| --- | --- | --- | --- | --- | --- | --- |
| **No** | | **VARIABLES** | **CODING CATEGORIES** | | **Remark** | |
| **101** | | Card No | _______________ | |  | |
| **102** | | Unique SAM Number | _______________ | |  | |
| **103** | | Age | ______(month) | |  | |
| **104** | | Sex | 1. male  2. female | |  | |
| **105** | | Residence | 1. urban  2. rural | |  | |
| **106** | | Referral source | 1. Health facility 2. Self‐referral | |  | |
| **107** | | Breast feeding on admission | 1.Yes  2.No | |  | |
| **108** | | Axillary temperature (°C) at admission | _______(°C) | |  | |
| **109** | | Vaccination status | 1. Fully vaccinated  2. Partially vaccinated  3. Not vaccinated | |  | |
| **110** | | Admission type | 1. new admission  2. re-admission | |  | |
| **111** | | Admission date | _______dd/-------------mm/-------------yyy | |  | |
| **Section 2. Anthropometric characteristics at admission** | | | | | | |
| **201** | Height | | | ------------(cm) | |  |
| **202** | Weight | | | -------------(kg) | |  |
| **203** | Wt/Ht | | | ---------------(%) | |  |
| **204** | MUAC | | | -------------(cm) | |  |
| **205** | Appetite at admission | | | 1. Failed appetite  2. Passed appetite | |  |
| **206** | Has history of Bottle feeding? | | | 1. yes  2. no | |  |
| **207** | Oedema | | | (0, +, ++, +++) | |  |
| **208** | Admission criteria | | | 1.Only edema(kwashiorkor)  2.Only wasting (W/H) (marasmus)  3.both edema and wasting  4.MUAC<11.5mm | |  |

| **SECTION 3. severe acute malnutrition diagnosis** | | | | | |
| --- | --- | --- | --- | --- | --- |
| **NO** | | **VARIABLES** | | **CODING CATEGORIES** | **Remark** |
| 301 | | Type of severe acute malnutrition | | 1. Marasmic  2. Kwashiorkor  3. Marasmic-kwashiorkor |  |
| 302 | | Major co-morbidities | | 1. TB  2. HIV  3. Malaria  4. Diarrhea  5. Pneumonia  6. Heart failure  7. Other(specify)________ |  |
| 303 | | Complication | | 1. Dehydration  2. Shock  3.Skin lesion  4.Sepsis  5.Hypoglycemia  6.Anemia  7.Others(specify)_______ |  |
| **Lab test results** | | | | | |
| **401** | Hgb | | -----------g/dl | |  |
| **402** | Malaria blood film | | 1. positive  2. Negative | |  |
| **403** | TB | | 1. Yes  2. No | |  |
| **404** | HIV test | | 1. Reactive  2. Non-reactive | |  |

| **SECTION 4. management of severe acute malnutrition** | | | | | | | | |
| --- | --- | --- | --- | --- | --- | --- | --- | --- |
| **Therapeutic diet** | | | | | | | | |
| **NO** | | | **VARIABLES** | | | **CODING CATEGORIES** | | **Remark** |
| 501 | | | Phases | | | 1. Phase 1  2. Transition Phase  3. Completed both phase | |  |
| 502 | | | Therapeutic foods administered | | | 1. F -75  2. F-100  3. RUTF (plumpnut) | |  |
| 503 | | | Milliliter/feed | | | 1. F-75 ---------ml/feed  2. F-100 --------- ml/feed | |  |
| 504 | | | Number of feeds per day | | | 1. F-75 ---------feed/day  2. F 100 ----------feed/day | |  |
| 505 | | | Additional food | | | 1. Porridge  2. Family meal | |  |
| 506 | | | Feed by | | | 1. Orally  2. By naso-gastric tube (NG –tube) | |  |
| **Section 5 Routine Medications** | | | | | | | | |
| 601 | | Vitamin A | | | 1. yes  2. no | | |  |
| 602 | | Folic acid | | | 1. yes  2. no | | |  |
| 603 | | Anti-malaria treatment | | | 1. yes  2. no | | |  |
| 604 | | De-worming | | | 1. yes  2. no | | |  |
| 605 | | Oral Antibiotic | | | 1. Amoxicillin  2. Other(specify)_______ _ | | |  |
| 606 | | Zinc | | | 1.yes  2.no | | |  |
| 607 | | Iron | | | 1.yes  2.no | | |  |
| **Special medications** | | | | | | | | |
| 701 | ReSomal | | | 1. yes  2. no | | |  | |
| 702 | IV fluids | | | 1. yes  2. no | | |  | |
| 703 | IV antibiotics | | | 1. yes  2. no | | |  | |
| 704 | Blood transfusion | | | 1. yes  2. no | | |  | |
| **Section 6 Discharge and outcome** | | | | | | | | |
| 801 | Discharged as | | | 1. recovered  2. defaulted  3. died  4. non-recovered | | | | |
| 802 | Discharge weight | | | _________kg | | | | |
| 803 | Discharge Wt/Ht | | | _________(%) | | | | |
| 804 | Discharge MUAC | | | ______(cm) | | | | |
| 805 | Length of stay | | | __________days | | | | |
| 806 | Discharged date | | | -------------dd/--------mm/----------yyy | | | | |
| 807 | Discharged time | | | -----------Am/Pm | | | | |
